# Barriers and opportunities for breast cancer organizations to focus on environmental health and disease prevention: a mixed-methods approach using website analyses, interviews, and focus groups

**DOI:** 10.1186/s12940-020-0570-7

**Published:** 2020-02-10

**Authors:** Jennifer Liss Ohayon, Eric Nost, Kami Silk, Michele Rakoff, Julia Green Brody

**Affiliations:** 1 Silent Spring Institute, 320 Nevada Street, Suite 302, Newton, MA 02460 USA; 2 University of Guelph, Guelph, Canada; 3 University of Delaware, Newark, USA; 4 Breast Cancer Care & Research Fund, Los Angeles, USA

**Keywords:** Breast cancer, Environmental health, Research translation, Activism, Risk factors, Environmental chemicals, Community outreach

## Abstract

**Background:**

Breast cancer is the most commonly diagnosed cancer among women worldwide and most cases are not due to high risk inherited genes. In response, breast cancer activists successfully advocated for innovative research on environmental chemical exposures as a possible cause. Since then, new evidence supports hypotheses that common industrial and consumer chemicals are linked to the disease, and expert panels recommend reducing exposures. We evaluated whether these research results and recommendations are translated back into the work of breast cancer organizations and what barriers and opportunities influence their ability to focus on environmental factors.

**Methods:**

We used a Python script to evaluate the frequency of environmental terms on the websites of 81 breast cancer organizations (> 14,000 associated URLs) and conducted two focus groups and 20 interviews with leaders of breast cancer organizations. We also analyzed the frequency of terms on two trusted, national cancer websites.

**Results:**

40% of organizations include information on environmental chemicals on their websites, but references are infrequent and rarely cite specific chemicals of concern. Most organizations (82%) discuss other risk factors such as exercise, diet, family history, or genetics. From interviews and focus groups, we identified four types of barriers to addressing environmental chemicals: 1) time and resource constraints, 2) limited knowledge of the state of the research and lack of access to experts, 3) difficulties with messaging, including concern that cultural and economic factors make it difficult for individuals to reduce their exposures, and 4) institutional obstacles, such as the downplaying of environmental risks by industry interests. Participants expressed the desire for easy-to-adopt educational programs and increased federal funding for scientist-advocate research partnerships.

**Conclusion:**

Our research underscores the need for environmental breast cancer experts and trusted cancer organizations to increase research translation activities so that breast cancer organizations can communicate new science on environmental factors in their online and in-person work. Moreover, our research highlights how most groups are focusing on providing resources to diagnosed women, including addressing problems with healthcare access, which displaces their ability to work on breast cancer prevention.

## Background

Breast cancer is the most commonly diagnosed cancer worldwide and in U.S. women aside from non-melanoma skin cancer, with states like Massachusetts declaring it an epidemic [1–3]. Moreover, incidence has been rising worldwide, with sharp increases in parts of the developing world since the 1970s [4]. Most cases are not a result of a high-risk genetic predisposition for the disease; for example, despite attention on the BRCA1 and BRCA2 genes, inherited mutations in these high-risk genes explain only 5–10% of breast cancers [5–7]. Well-established modifiable risk factors for breast cancer include menopausal hormone therapy, reproductive history, lack of physical activity, weight gain after menopause, and alcohol consumption, although the strength of association varies based on disease subtype or life stage [8–10]. While research often emphasizes pharmaceutical and lifestyle-related factors as important for reducing risk, environmental exposures can also be critical targets for breast cancer prevention. Laboratory studies indicate that many common chemicals, including those in air and water pollution and personal care products, plastics, and home furnishings, are mammary gland carcinogens or endocrine disrupting compounds (EDCs) that may increase breast cancer risk [11–15], while epidemiological studies find heightened breast cancer risk associated with solvents, polycyclic aromatic hydrocarbons (PAHs) and air pollution, dichlorodiphenyltrichloroethane (DDT), dioxins, and other exposures [16, 17]. The importance of environmental chemicals as a target for breast cancer prevention is underscored in key expert reports, including the 2007 California Breast Cancer Research Program report *Identifying Gaps In Breast Cancer Research* [18], 2010 President’s Cancer Panel report *Reducing Environmental Cancer Risk: What We Can Do Now* [19], the 2012 Institute of Medicine report *Breast Cancer and the Environment: A Life Course Approach* [20], and the 2013 Interagency Breast Cancer and Environmental Research Coordinating Committee (IBCERCC) report *Breast Cancer and the Environment: Prioritizing Prevention* [21]*.*

Historically, breast cancer activists catalyzed much of the newer research on the links between breast cancer and pollutants, channeling their frustrations that cancer research was not addressing their questions about environmental factors and prevention [22]. For example, breast cancer activists won federal legislation that mandated the Long Island Breast Cancer Study Project [23], which was the first large-scale research on breast cancer and the environment and encompassed more than 10 studies totaling over $30 million in research funds [24]. At the same time, breast cancer activists won a 1 million dollar annual state appropriation in Massachusetts to fund the Cape Cod Breast Cancer and Environment Study. This funding supported the grassroots Massachusetts Breast Cancer Coalition in establishing Silent Spring Institute in 1994 as an independent research group dedicated to breast cancer and environment research [25]. Breast cancer activists also achieved the creation of a multicenter National Institutes of Health program of research into breast cancer and environmental factors over the life course [22, 26], referred to as the Breast Cancer and the Environment Research Center and later the Breast Cancer and the Environment Research Program (BCERP). In all of these cases, breast cancer activists have not just initiated new federal and state legislation but have been important contributors to research design and implementation [22]. In addition, breast cancer advocacy groups, such as the National Breast Cancer Coalition, were crucial to the creation of significant federal reports, albeit in the case of the IBCERCC report, some were angered that federal funds shifted from an original intent to support new research to funding a report [27].

While activists expanded the scope of the research agenda, the overwhelming majority of breast cancer funding and research remains directed at diagnosis and treatment, rather than at environmental health and prevention. In its 2013 report, IBERCC noted that, at most, 10 to 11% of breast cancer projects funded by the National Institutes of Health (NIH) and the U.S. Department of Defense (DOD) focused on environmental health [21]; recent estimates are similar and indicate that of the approximate $2 billion spent on breast cancer research each year, less than 10% is directed to prevention research [28]. Nevertheless, several important initiatives exist to advance research on the environment and breast cancer, including the National Institute of Environmental Health Sciences (NIEHS)/National Cancer Institute’s (NCI) BCERP. In addition, NIEHS’ strategic plan 2018–2023 focuses on the importance of translating the findings that emerge from these federal research programs. For example, Theme 2 of the strategic plan states “Efforts will continue in promoting research findings to networks of scientists, community advocates, educators, healthcare providers, and public health officials, who can translate evidence into credible and understandable information and actions that individuals and communities can use to decrease their risk, prevent harm, and improve their health” [29].

Given the role of breast cancer activists in stimulating environmental research, and the NIEHS goal to translate research into public health, we were interested in evaluating whether research from federally-funded and other research programs is reflected in the communications and day-to-day work of breast cancer advocates. As such, we used website analysis, focus groups, and interviews with activist leaders to learn how environmental research is currently included in their communications, and to understand the barriers and opportunities to doing so in the future. For this study, our definition of environment focuses on chemical agents that people are exposed to through industrial and vehicular pollutants, consumer products, and pesticides. Our work is the first to systematically evaluate the online contributions of breast cancer organizations to prevention-based efforts. Results will inform opportunities for researchers to better translate their work to organizations that are doing on-the-ground outreach and community engagement work.

## Data collection and methods

We used a mixed-methods approach, where we a) analyzed the websites of 81 U.S. breast cancer organizations for content that addresses environmental and other risk factors, b) convened two focus groups and conducted 20 interviews with leaders of breast cancer advocacy organizations, and c) analyzed the breast cancer risk factor pages on the websites of two national cancer organizations, NCI and the American Cancer Society (ACS). We selected NCI and ACS for study, because they are both prominent cancer organizations that serve as resources for smaller groups.

### Website analysis

We used an automated, large-scale approach involving scraping terms from websites in order to characterize how 81 U.S. breast cancer organizations and two prominent national cancer organizations, ACS and NCI, incorporate information on risk factors.

To select breast cancer organizations’ websites for review, we began from a list of contacts developed by Silent Spring Institute in 2008 through systematic internet searches and snowball sampling of breast cancer advocacy networks. We returned to these websites to identify organizations that were still active. In addition, we included local, regional, and national breast cancer advocacy and research organizations identified on the websites of the National Accreditation Program for Breast Centers and the National Comprehensive Cancer Network, and we added organizations listed multiple times on the resource pages of breast cancer organizations in our original list. Our final database includes organizations active in 24 states, plus 35 national organizations (see Additional file 1: Table S1). We visited websites to categorize groups as either a) national groups or b) local or regional for later analysis.

We used Python programming language scripts to “crawl” and “scrape” the websites of breast cancer advocacy organizations. Web crawling scripts like ours start by accessing each homepage - e.g. www.example.com - and collecting all of the links that point to other example.com pages (e.g. www.example.com/page-one, www.example.com/page-two, etc.). In this way, the script “crawls” through the website to produce a list of available and linked-to pages. In a second step, we “scraped,” or extracted, all of the content on each page in order to quantify the presence of key terms associated with environmental and other risk factors. Before scraping pages, we cleaned up the crawling output by removing non-relevant content, such as broken links, sponsor pages, pages devoted to selling products, and message boards and forum pages. We excluded pages in Spanish, because we scraped pages for English terms and equivalent Spanish terms would not be systematically represented. Pages in Spanish belonged primarily to the national breast cancer organizations Living Beyond Breast Cancer, Breastcancer.org, and Komen, as well as smaller organizations dedicated to serving Latinx communities, namely Circula de Vida and Latinas Contra Cancer. For one organization (Huntington Breast Cancer Action Coalition), we combined the main website (http://www.hbcac.org) and their educational campaign website (Prevention is the Cure; http://www.preventionisthecure.org/). One organization, Imaginis, compiled resources relating to women’s health in general so we subset the crawled URLs to just the pages addressing breast cancer.

For the National Cancer Institute and American Cancer Society, we selected the main pages addressing breast cancer by identifying URLs in the crawled output that contain the term “breast” and visiting these flagged URLs to verify they were focused on breast cancer. Our sampling approach does not capture general pages that discuss risk factors for cancer, but rather should be viewed as a representative sample of pages focused on breast cancer.

To count terms, we first used the Internet Archive’s Wayback Machine to archive “snapshots” of pages in mid-November 2018 for 81 breast cancer organizations and in mid-December 2018 for NCI and ACS. For a small subset of URLs that we could not archive, we sought an existing version in the Wayback Machine for the comparable time period. If a snapshot was not available, we accessed URLs as they existed live in mid-November for the breast cancer organizations and mid-December for NCI and ACS. We then scraped the websites for 52 terms associated with risk factors for breast cancer (see Table 1). The terms “environment,” “toxic,” and “chemical” were not included, because they sometimes appeared on websites in a context unrelated to environmental exposures (e.g., the term “chemical” can occur in a discussion of chemotherapy, and similarly “toxic” can refer to effects of radiation or chemotherapy treatments). Likewise, “hormone therapy” was omitted, because it is often used in the context of a breast cancer treatment, but hormone replacement therapy (HRT) was included as it is used more frequently to refer to a risk factor for breast cancer.

**Table 1:** How frequently topics are mentioned on the websites of breast cancer organizations. N (Total organizations) = 81

Factor	Type of Factor	Representative terms analyzed on websites	Percent (%) of organizations that mention factor (N in brackets)	Number of mentions across organizations
Exercise	Other	Exercise, physical activity, physically active	74 (60)	2796
Family history	Other	Family history	67 (54)	1167
Diet	Other	Diet	59 (48)	1514
Genetics	Other	Genetics, BRAC1, BRAC2	52 (42)	1257
Alcohol	Other	Alcohol	48 (39)	722
Breast density	Other	Breast density, dense breasts	44 (36)	542
Body weight	Other	Overweight, obese	36 (29)	456
Hormone replacement therapy	Other	HRT	30 (24)	800
Pesticides	Specific environmental	Pesticide(s)	30 (24)	565
Bisphenols	Specific environmental	BPA, bisphenol(s)	25 (20)	1582
Contamination	General environmental	Contaminant(s), contamination	25 (20)	517
Pollution	General environmental	Pollution, pollutant(s)	25 (20)	644
Birth control	Other	Birth control, oral contraceptives	23 (19)	398
Parabens	Specific environmental	Paraben(s)	23 (19)	419
Endocrine disrupting chemicals	Specific environmental	Endocrine disruptor(s), endocrine disrupting chemical(s), hormone disrupting chemical(s)	21 (17)	382
Phthalates	Specific environmental	Phthalate(s)	17 (14)	546
Diethylstilbestrol	Other	Diethylstilbestrol, DES	16 (13)	253
The precautionary principle	General environmental	Precautionary principle	15 (12)	142
Flame retardants	Specific environmental	Flame retardant(s)	12 (10)	1074
Air pollution	Specific environmental	Air pollution	11 (9)	80
Polycyclic aromatic hydrocarbons	Specific environmental	PAH(s)	11 (9)	104
PFAS	Specific environmental	PFOA, PFOS, PFAS, PFC(s), perfluorinated chemicals	11 (9)	230
Oxybenzone	Specific environmental	Oxybenzone	7 (6)	18

After deleting non-relevant pages and excluding 843 URLs (distributed across 48 organizations) that the script was unable to decode, we were left with a final database representing 14,087 webpages for breast cancer organizations, as well as 463 pages for NCI and 348 pages for ACS. We analyzed these results in R programming language version 3.5.0. During analysis, we grouped together similar terms, for example “pollution,” “pollutants,” and “pollutant” (see Table 1 for groupings). We also categorized terms as relating to “general environmental factors” (e.g., pollution), “specific environmental factors” (e.g., phthalates and flame retardants), or “other factors” which encompassed risks related to lifestyle (e.g., diet and exercise), genetics, body size, family history, or pharmaceutical use (e.g., birth control).

For breast cancer organizations, we analyzed the total number of times that terms were referenced across all the websites and the percentage of organizations that referenced the terms in order to get an overview of their priorities. For NCI and ACS, we analyzed the number of times that terms were referenced and also visited their websites to qualitatively assess their discussion of risk factors for breast cancer.

### Focus groups and interviews

We conducted two focus groups (15 participants) and 20 semi-structured interviews with representatives from U.S. breast cancer organizations between November 2016–December 2018 to understand the programmatic priorities of grassroots breast cancer organizations and the potential for them to incorporate information on prevention.

We recruited participants by personalized emails. We randomly sampled potential participants from our database of breast cancer organizations described above. Additional participants were recruited using snowball sampling methods, where colleagues working with breast cancer organizations recommended participants, and targeted sampling from our database to include breast cancer organizations that serve diverse ethnic, racial, and linguistic populations.

Our participants were geographically diverse, representing organizations based in 16 U.S. states and including 7 national organizations. Five participants were from breast cancer organizations that primarily serve women of color who face disparities in access to information and health services (i.e., Latinx and African American women) and one was from an organization serving young adults diagnosed with breast cancer. All participants except for three were also from organizations that also had their websites analyzed. Participants represented organizations oriented around services for members, educational outreach, advocacy and lobbying, and research. All were women, and they held various positions including executive director, president, program manager, outreach and education director, research scientist, mission coordinator, and regional liaison.

Focus groups lasted approximately 60 min and were conducted online using Zoom Video Conferencing software, and sound was recorded and transcribed. We used video conferencing, rather than in-person focus groups, to include a broader geographic range of participation. Our experience with online focus groups was that there was meaningful interaction among participants and that participants built on each other’s comments on par with focus groups that we’ve led in-person (this was aided by participants having their names displayed in Zoom and selecting the “gallery” view option so that all faces were visible at the same size on the screen). We ensured all participants tested out their video and sound beforehand so that there was no troubleshooting during the focus groups. The focus group moderator guide asked if the participant’s organization targeted environmental risks for breast cancer and inquired about barriers to considering these factors as part of their organization’s current scope. Questions also gauged participants’ pre-existing knowledge and perspectives on environmental factors, current sources for information on breast cancer, and actions they would like government and industry to take to reduce breast cancer risk. Focus groups were recorded with permission and then transcribed.

Interviews were conducted by phone and lasted approximately 45 min. We asked interviewees about the areas of focus for their organization’s work, whether any work has centered on breast cancer prevention and/or environmental factors, and barriers for considering environmental factors as part of their organization’s scope.

Focus group transcripts were coded and analyzed using Dedoose, a qualitative data management and analysis tool. Our coding approach primarily consisted of a priori codes based on the specific questions in our moderator guide, as well as on the broader categories and conceptual themes addressed by this study. The interviews were meant to complement focus group data and observe whether parallel themes emerged in one-on-one conversations. Most of the phone interviews were not recorded and instead detailed notes were taken that summarized the main topics of conversation and paraphrased important statements. We then returned to interviewees to verify the accuracy of quotes for ones that we included in the manuscript. Moderator guide and interview questions are available from authors upon request.

## Results

Website analyses showed that while many organizations include information on environmental factors on their websites, mentions are infrequent and limited with respect to information on specific chemicals of concern; moreover, existing online messages focus more on lifestyle factors and genetics. Our interviews and focus groups revealed the main barriers to providing more information on environmental factors.

### Websites of breast cancer organizations have limited discussions of environmental factors, with more frequent mentions of lifestyle and genetic risk factors

**Fig. 1:**
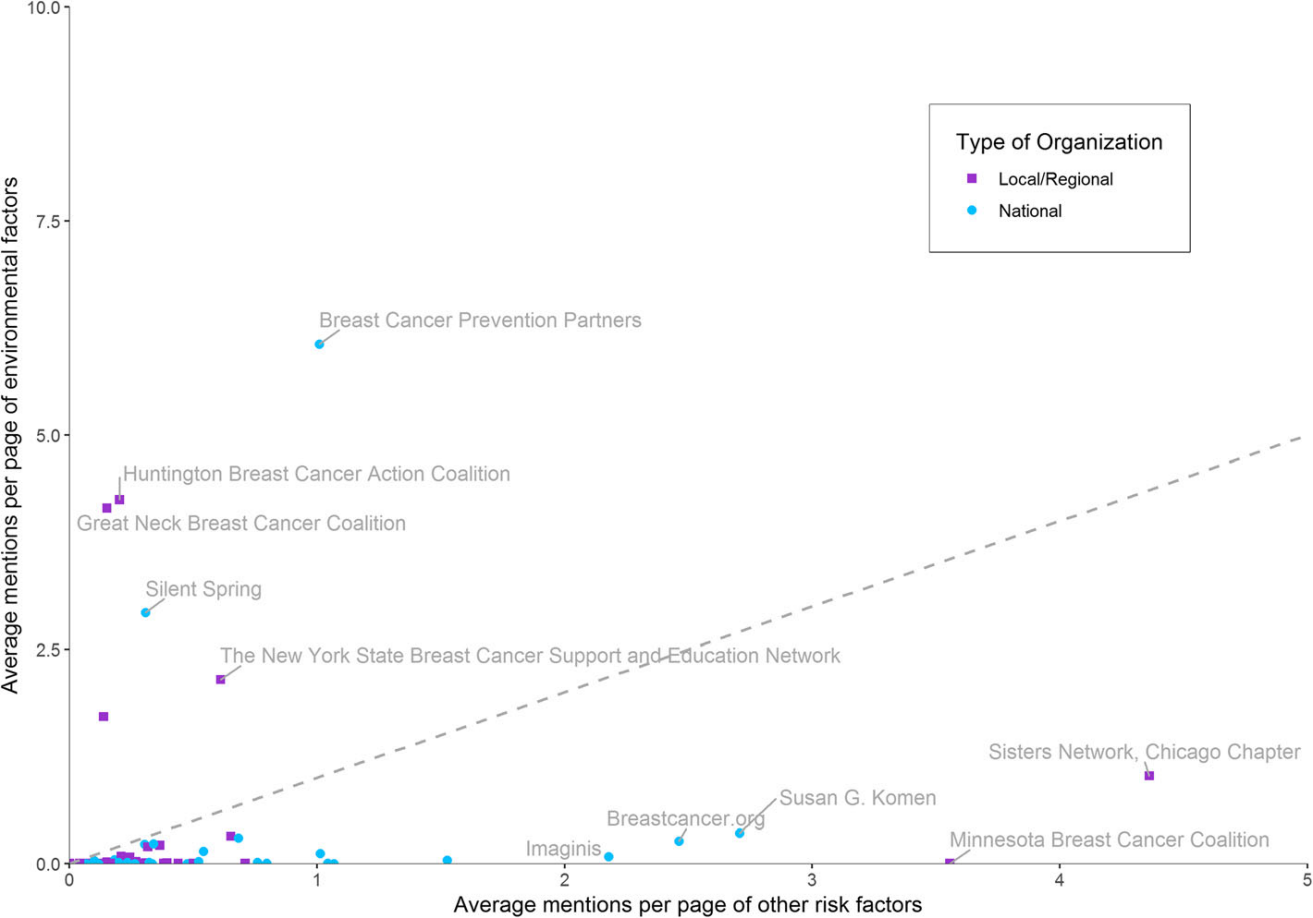
Average mentions per page of environmental topics versus other risk factors, such as lifestyle or genetic, on organizations’ websites. Organizations that are primarily national in scope are represented in blue circles and local or regional organizations are represented in purple squares. Dashed line indicates equal average usage of both environmental and non-environmental terms. N (Total organizations) =81

We analyzed terms on 14,087 URLs belonging to 81 breast cancer organizations (websites averaged 174 associated URLs, with a range from 3 to 894). We found 31% of the organizations made a general mention of the environment (i.e.,, mentioned general terms such as “contamination” or “pollution,” rather than chemical-specific factors; please see Table 1 for terms categorized as “general environmental”), 40% mentioned a specific class of chemicals that may act as breast carcinogens or endocrine disrupting compounds, and 82% mentioned a risk factor not related to environmental exposures. While the majority of organizations mentioned either an environmental, lifestyle, or genetic risk factor on their websites, it was rare that website content discussed risk factors at length based on our term scraping analysis; most organizations mention these topics less than once per page on average, with references to environmental factors in particular clustering at or close to zero for many websites (Fig. 1).

The most frequently mentioned risk factors were unrelated to environmental exposures (Table 1). Nearly three-quarters of organizations discuss exercise; family history, diet and genetics were also frequent terms (discussed by > 50% of the organizations). For specific environmental chemicals, pesticides appeared most frequently (30% of organizations mentioned the term) and oxybenzone was the least mentioned (7% of organizations mentioned the term). The terms “air pollution” and “PAH(s)”- a common air pollutant as well as constituent in tobacco smoke and food cooked at high temperature - were each mentioned by 11% of organizations. Flame retardants were only discussed by 12% of organizations, but a few of these organizations, primarily Silent Spring Institute and, to a lesser extent the Massachusetts Breast Cancer Coalition and Breast Cancer Prevention Partners, frequently discussed this class of chemicals on their websites and hence the total number of mentions is relatively high (i.e., the total mentions of flame retardants was 1074 times, which consisted of 911 times by Silent Spring, 111 times by Massachusetts Breast Cancer Coalition, and 32 times by Breast Cancer Prevention Partners; the seven remaining organizations that referenced flame retardants mentioned this class of chemicals less frequently). Similarly, while a quarter of organizations reference bisphenols, the high number of total mentions is driven largely by a few organizations, Breastcancer.org, Breast Cancer Prevention Partners, and the Massachusetts Breast Cancer Coalition, having in-depth coverage of Bisphenol A on their websites (Table 1).

When comparing the number of times on average that organizations mentioned environmental chemicals versus other risk factors, we found that organizations tended to specialize in one or the other (Fig. 1). In general, organizations whose websites contained many mentions of environmental chemicals had few references to other types of risk factors and vice versa. Both local/regional and national organizations focused on environmental factors; for example, websites of local groups (such as the Massachusetts Breast Cancer Coalition and Huntington Breast Cancer Action Coalition) and national groups (such as the Breast Cancer Prevention Partners and Silent Spring Institute) contained extensive information on environmental factors.

### Information from NCI and ACS

We also assessed the websites of NCI (www.cancer.gov) and ACS (www.cancer.org) as both are national groups that are considered key sources for information on breast cancer; ACS in particular was referenced during focus groups (described in greater detail below) as an organization that local breast cancer organizations rely on for information to share with their members.

A qualitative analysis of their websites found that both NCI and ACS list environmental carcinogens that are not breast-cancer specific, referencing classifications by the National Toxicology Program and International Agency for Research on Cancer. Moreover, both discuss exogenous hormones that influence breast cancer, specifically the pharmaceuticals DES and hormone replacement therapy. While there is some general discussion of environmental chemicals and cancer on their websites, there is little information on the sections of their websites that are breast cancer-specific. A quantitative term analysis of breast cancer-specific pages on NCI and ACS’s websites demonstrated that genetic and family history risk factors and lifestyle risk factors, such as exercise and diet, are discussed more frequently than environmental factors (Table 2). Breast cancer pages on the NCI site include no specific mentions of “endocrine disrupting chemicals” or “endocrine disruptors” or of air pollution or PAHs, and breast cancer pages on the ACS site include one mention of endocrine disruptors and no mentions of air pollution or PAHs. Both NCI and ACS, however, do briefly mention environmental chemicals can have “estrogenic” or “estrogen-like” properties on their breast cancer pages. Both websites state that there is little evidence to suggest that environmental chemicals increase cancer risk.

**Table 2:** Discussion of environmental factors on breast cancer pages of National Cancer Institute and American Cancer Society websites (463 NCI URLs and 348 ACS URLs that specifically address breast cancer rather than the websites in full)

Risk Factor	Representative terms analyzed on websites	Number of mentions on NCI website	Number of mentions on ACS website
Family history	Family history	249	74
Breast density	Breast density, dense breasts	171	66
Genetics	Genetics, BRAC1, BRAC2	140	67
Exercise	Exercise, physical activity, physically active	96	340
Hormone replacement therapy	HRT	37	100
Alcohol	Alcohol	34	69
Birth control	Birth control, oral contraceptives	32	27
Diet	Diet	28	172
Body weight	Overweight, obese	21	77
Contamination	Contaminant(s), contamination	9	0
Pesticides	Pesticide(s)	2	1
Diethylstilbestrol	Diethylstilbestrol, DES	2	5
Bisphenols	BPA, bisphenol(s)	0	0
Pollution	Pollution, pollutant(s)	0	1
Parabens	Paraben(s)	0	25
Endocrine disrupting chemicals	Endocrine disruptor(s), endocrine disrupting chemical (s), hormone disrupting chemical(s)	0	1
Phthalates	Phthalate(s)	0	0
The precautionary principle	Precautionary principle	0	0
Flame retardants	Flame retardant(s)	0	0
Air pollution	Air pollution	0	0
Polycyclic aromatic hydrocarbons	PAH(s)	0	0
PFAS	PFOA, PFOS, PFAS, PFC(s), perfluorinated chemicals	0	0
Oxybenzone	Oxybenzone	0	0

NCI’s Breast Cancer Prevention (PDQ) cancer information summary, for example, outlines general information about breast cancer and strategies for avoiding risk factors. While the PDQ is reviewed and updated by a board that is editorially independent from NCI, it is the main resource outlining the evidence on breast cancer risk factors that NCI provides for patients and health care professionals. For the patient PDQ, the section on the environment only states “Studies have not proven that being exposed to certain substances in the environment, such as chemicals, increases the risk of breast cancer” [30]. The PDQ that is aimed at health care professionals was recently updated to cite two reviews of epidemiological studies of environmental chemicals and breast cancer [16, 17], both of which highlight evidence for chemical links to the disease. However, the powerful 50-year Child Health and Development Studies cohort is not cited, and results showing increased risk in both mothers and daughters exposed to DDT is described as conflicting, without noting the investigators’ analysis that associations with different components of DDT in the mothers and daughters are due to timing of exposure [31]. The section states that “Overall, the epidemiological and animal study evidence that support an association between breast cancer and specific environmental exposures is generally weak,” while not describing or citing the animal or mechanistic evidence [32].

ACS’s general website contains information on environmental chemicals and links to external carcinogens lists from the International Agency for Research on Cancer (IARC) and the National Toxicology Program. When restricting the ACS’s website to sections that focus specifically on breast cancer, environmental chemicals are briefly discussed and classified as “Factors with Unclear Effects on Breast Cancer Risk,” with the website stating

Compounds in the environment that have estrogen-like properties are of special interest. For example, substances found in some plastics, certain cosmetics and personal care products, pesticides, and PCBs (polychlorinated biphenyls) seem to have such properties. In theory, these could affect breast cancer risk. This issue causes a great deal of public concern, but at this time research does not show a clear link between breast cancer risk and exposure to these substances [33].

Both websites have lifestyle-related tips, such as exercising and reducing risk factors such as drinking alcohol, as key recommendations for reducing overall cancer risk.

### Leaders of breast cancer advocacy organizations are concerned about environmental links

Focus groups and interviews gave further insights into how breast cancer organizations across the country have incorporated environmental topics into their work. While the majority of U.S. breast cancer organizations do not emphasize environmental factors when presenting information online, almost all focus group participants and many of the interviewees were concerned about possible links between environmental chemicals and breast cancer. They were especially concerned about pesticides, air pollution, and consumer products. For example, one focus group participant stated,

I live in a really agriculture rich rural area and I’m confident that a lot of our cancer diagnoses, not just for breast cancer but for many, many kinds of cancer, are directly related to the amount of chemicals that are being sprayed.

Another followed up, saying,

I’m extremely concerned as well about what we’re using, and while I have to admit that I’ve not made any big changes as some of you all have by going to more natural products, I do read a lot more labels now … So I would like to get to a point where we wouldn’t have to worry about the dangers of the products that we use routinely in our home.

Despite participants’ concerns, the organizations they worked for and led, were typically oriented more towards providing services for women diagnosed with breast cancer, including providing childcare and transportation to medical care and screening, helping uninsured or underinsured women access health care, or encouraging women to get screened.

While they were not representative of our overall sample, several organizations included in our focus groups and interviews are dedicated to supporting scientific research and advocacy targeting the environmental causes of breast cancer. These groups focused on community education programs and state and federal lobbying to increase environmental research funding or reduce exposures through policy change. For example, one group, concerned about possible links between high rates of breast cancer in their community and drinking water contaminants, helped create the pesticide registry in New York State. Another group supported a successful bill to reduce the use of toxic flame retardants in public spaces. These groups were often founded by survivors of breast cancer with the goal of preventing others from suffering a similar diagnosis. As one interviewee stated,

Obviously you want to take care of people who are sick, and we have a need for new directions for treatment, but if we can keep people from getting sick in the first place that’s a lot better. The costs of breast cancer go beyond financial and include suffering from the treatments, family disruption, job disruption, uncertainty. If we can stop people from getting sick we should absolutely be doing the work to keep that from happening.

### Barriers to addressing environmental factors

While many focus group and interview participants expressed concerns about environmental factors, they also enumerated multiple barriers that limit their ability to incorporate the environment and prevention in their work. Barriers fell into four categories: 1) time and resource constraints, 2) limited knowledge of the state of the research and lack of access to experts in the field, 3) difficulties with developing messaging that is, for example, sensitive to cultural and economic factors that might make it difficult for individuals to adopt recommendations, and 4) institutional obstacles, such as the downplaying of environmental risks by industry interests.

#### Time and resource constraints

In both focus groups and several interviews, representatives of breast cancer organizations said they would like to do more educational and advocacy work, but their organizations are small and resource-limited, so they direct most of their staff time or resources to providing services for their members. As one stated, “we don’t have, again, that dedicated staff or the time to really focus, and we have multiple focuses already within our main coalition.” Another said,

I’m more interested in research and interested in lobbying to save DOD [Department of Defense] funding for breast cancer research. And I don’t have the time or personnel or energy to do “What is the next step?” …. Most of the time, participants are looking for the next movement and they look to me to do it … I feel sometimes that I should have the next step, but haven’t really had the time or the energy to do that.

Beyond feeling limited in terms of staff time, four participants cited that it can be difficult to find funding to support that type of outreach and research. Some said that much of their organization’s work is done by volunteers, and speculated that their volunteers might lack interest in working on environmental issues.

#### Uncertain science and limited knowledge of existing research

While some participants were well-informed about environmental factors for breast cancer, focus groups and interviews revealed two key scientific barriers to communicating information on environmental chemicals. Firstly, breast cancer advocates sometimes viewed the scientific evidence as uncertain and were thus hesitant to communicate information about it; these participants wanted to do educational outreach only on risk factors that are “supported by science,” “trusted,” or “valid.” For them, the perception is that lifestyle-related risk factors, such as smoking, exercise, and diet, are scientifically established, but significant gaps in knowledge and a lack of a conclusive body of evidence for environmental chemicals make it difficult to speak confidently with their members. As one focus group participant stated, “I think that what’s really challenging about going out and talking with people in the community is that we don’t really know.”

Secondly, many advocates lacked confidence in the depth of their expertise about environmental chemicals and were unaware of the science that does exist. As one interviewee stated:

It’s a tricky balance at the end of the day because organizations want to make sure what they say is true and evidenced-based and if someone inquiries about something that is on their website that they can talk about it. And if they don’t feel like they have enough data, then they’d potentially rather not open the door for fear they aren’t knowledgeable enough. So it’s easier to just avoid discussing cancer and the environment.

#### Messaging to avoid hopelessness or blame

Participants discussed struggles with how to adapt messaging to reach populations where there might be a cultural stigma around breast cancer in general or where economic constraints might make it difficult for individuals and communities to adopt exposure reduction recommendations. For example, one stated.

I work with women, Latinas who are already diagnosed and the majority of our clients are low-income … So a lot of them are struggling with issues that are more important to them than what causes breast cancer, like trying to feed their families and trying to keep their homes or their apartments, or whatever. Although I bring all that information [on environmental factors] to groups, it’s really difficult to tell low-income people, ‘Eat healthier, buy foods without pesticides.’ Well, that’s easy to say, but it’s not so easy for people who are already struggling financially.

A participant followed up on this sentiment by saying, “It’s very challenging... You don’t want to make people paranoid about where they’re stuck living because of their financial situation.” Another said, “You can’t shop your way out of this problem. People don’t have the resources to do that. Which is why the legislative work is so important as well.”

Furthermore, participants expressed reluctance to speak about prevention-based approaches out of a more general concern that people will feel responsibility for their diagnosis (i.e., that they did something “wrong”). As one participant said, “[it’s] difficult to message because we’re always trying to not blame the victim, and it can come across that way when you’re talking about environmental factors. And so, it’s very tricky messaging for us … we’re always trying to think of how do we message this in a positive way?” In contrast, one interviewee mentioned that it’s important to focus on environmental risks so that breast cancer survivors do not blame themselves (e.g., their lifestyles) for their diagnosis, but recognize there are larger structural issues that put women’s health at risk.

One participant mentioned that prevention messages can be a “hard sell” among their constituents and communities. As she stated,

Unfortunately, I think trying to convince women or women and men who have not had the disease, or have not had it in their family, is a bit more difficult because as we all know we kind of put things off until we have to, and then when you get a major health scare, that can be very compelling as a reason to start looking into healthier behaviors, or avoiding certain chemicals and things …. We have to find a way to make it hit home harder to people who have not yet been through it.

Three participants from organizations that disseminate prevention-based messages, however, mentioned that their communities respond well to research-based strategies on how to reduce risks and thus think it is important to provide this type of information.

#### Institutional obstacles

Focus groups participants had a lively conservation about what they perceived to be disproportionate industry influence on research and regulations. They described feeling overwhelmed by the massive industry efforts that undermine the significance of environmental factors. As one participant stated,

You’ve got these major corporations that are paying zillions of dollars to get their cancer-causing products out there, and they’re going to do anything they can to discredit and squash the efforts of anyone who’s trying to rise up against them. And with the funding that they have they can be far more successful than a volunteer advocacy-organization based in rural [state omitted]...

In addition to increased government funding for breast cancer research, participants expressed the wish that federal public health agencies such as the EPA, FDA, and OSHA would do a better job regulating companies and restricting harmful products from hitting the marketplace. As one interviewee stated, “If we don’t change policies at the same time we are funding research, we don’t change what counts …”.

Participants also brought up a general lack of institutional leadership on the environment and breast cancer. One focus group participant stated, “Most of the conferences that I go to are all about treating about the disease … I’m wondering what the barriers are for the large institutions or the centers of excellence or the universities or whatever, why is that on a backburner?” One interviewee emphasized how this absence of leadership interacts with a lack of knowledge and inclination on the part of individual organizations to discuss environmental factors:

Because organizations don’t have a lot of concrete information as it relates to cancer and the environment, people tend to not want to talk about it because it will open up to all these other questions that they don’t have answers to... That’s what I’ve heard people at different organizations say as to why they won’t put environmental risk factors, etc. on their websites or on the agenda at conferences. There are a lot of breast cancer conferences, whether they are ones that organizations do for their particular constituents, or look at San Antonio, the environment doesn’t get center stage there. So they figure if San Antonio is the largest breast cancer conference and they aren’t tackling it, then why should we?

### Opportunities to better address environmental factors

When we asked how breast cancer organizations could better incorporate environmental factors into their work, some representatives wanted increased online and in-person access to experts who could answer their questions and speak to their members (e.g., through podcasts, videos, webinars, and workshops). Others communicated the need for an educational program on the environment and breast cancer they could easily adopt (e.g., booklets they could share while tabling). As one stated, “… if there was a program even on the environment that was already structured that [my organization] could just do, but I didn’t have to work at putting it all together, that would be wonderful.” Another echoed this saying,

For us, because we are volunteer survivor run, we’re limited as I’ve heard multiple responses already. What would help is programs that are easy to promote and for us to get our hands on in order to put them into the community … We are focused mainly on the survivorship portion. However, if there was an opportunity to tap into quality programs that were easy for us to understand and roll out with the programs that we’re already rolling out, that would be fine.

One participant, however, pointed out the importance of ensuring that resources are tailored for particular communities. As she stated,

If people have spent time and developed a model and it was successful, then they think it can be replicated everywhere with the same success, and that’s not always the case when you are dealing with more diverse communities. Some tweaking is essential so you have the right messaging, visuals, and are speaking the correct language.

In particular, participants said that they would like more resources in Spanish and other languages to share with their communities. As one stated,

That would really help. Because my groups are struggling with a lot of things in their lives, but they do want information... So, I could go in and explain it to them, which is what I usually do, because there’s not a whole lot of Spanish out there. But it would be nice to have these materials in Spanish.

Our website analysis did identify that some organizations, namely Living Beyond Breast Cancer, Breastcancer.org, Komen, Latinas Contra Cancer, and Circula de Vida, do have sections with general information on breast cancer (i.e., not necessarily related to environmental factors) in Spanish.

While participants expressed the need for more resources on the environment and breast cancer, participants in both focus groups and several of the interviews did reference organizations that they look towards as providing authoritative information on general risk factors for the disease. These included individual physicians and researchers, Susan G. Komen for the Cure, the American Cancer Society, Breastcancer.org, National Breast Cancer Coalition, Living Beyond Breast Cancer, and Breast Cancer Action (only Breast Cancer Action was referenced as authoritative in the context of environmental factors specifically). While a participant made a general reference to the NIH as a source of information, only one participant who received previous funding from BCERP specifically mentioned this NIEHS- NCI research program.

One focus group participant underscored that national organizations, such as Breast Cancer Action, can also serve the critical role of alerting smaller organizations to important political and legislative developments. As she stated,

Thankfully you’ve got a few really wholehearted grassroots organizations that are focused on this issue and bringing these things to our attention, but as individuals, or even just a few, a handful of volunteers running an organization, it’s hard to be abreast or be vigilant of every bill that’s coming across the Congress floor. It’s nice that when you do have organizations that you can plug into that are being the watchdog.

Focus group participants and interviewees also emphasized the importance of funding for federal programs that investigate risk factors for breast cancer by pairing researchers with advocates. In both focus groups, participants wanted funding to be maintained for the Department of Defense’s breast cancer research program (it was referenced four times in one focus group alone).

## Discussion

Breast cancer organizations can be critical actors when it comes to preventing the disease; hundreds of local and national breast cancer organizations exist in the U.S., making them an important vehicle for reaching patients and communities and shaping national discourse and policies on breast cancer. Breast cancer organizations, however, are an untapped resource for informing people about the large body of information on the environment and breast cancer. Over 80% of organizations surveyed in our website analyses mentioned a lifestyle or genetic risk factor on their website. There was less information, however, related to environmental health as only 40% of organizations mentioned a specific environmental factor. As organizations are already incorporating risk factors into the information they provide to their communities online, there is great potential to enrich outreach efforts with up-to-date and meaningful information about environmental chemicals and a broadened emphasis beyond lifestyle and genetic risk factors.

Representatives of breast cancer organizations wanted to provide responsible, culturally-sensitive information on disease prevention. However, they were reluctant to speak to their communities because of a lack of personal knowledge about environmental links or because they believe the research to be inconclusive. This likely also translated to a paucity of information about environmental chemicals on their websites. Advocates were largely unaware of the extensive scientific literature and statements by expert panels and professional organizations, including IBCERCC, the Institute of Medicine, and the President’s Cancer Panel, which conclude that evidence is sufficient for taking preventative actions to reduce exposures to chemicals with biologically plausible links to breast cancer [14, 16, 19–21]. It is thus critical that the research community and major cancer organizations work to provide better resources to breast cancer organizations on disease prevention; these resources should both highlight the current research on breast cancer and the environment, as well as explain the role of precautionary actions when the science is uncertain [34, 35]. Other research suggests that much of the existing online information on the topic is low quality, non-accessible, or suggests no association between environmental exposures and breast cancer [36], further signaling a need for increased research translation from federally funded programs and the research community.

In particular, federally-funded breast cancer and environment programs, such as the NIH BCERP and DOD’s Breast Cancer Research Program, can be important sources of information for breast cancer organizations. BCERP in particular has a website (https://bcerp.org/) that offers meaningful information on breast cancer and the environment and a research translation program that partners scientists, communication researchers, and breast cancer advocates. We interviewed an advocate partner who discussed her trust in BCERP materials, her increased confidence in communicating information about environmental chemicals to the public, and her belief that her years working with the program “are not going to waste.” Nevertheless our work found that BCERP resources have not been taken up by major websites, such as the NCI PDQ or ACS, and are not reaching many breast cancer advocates. It is thus important that research results continue to be actively and broadly translated to breast cancer organizations that interface frequently with the public, including with those who are not affiliated with these research programs. For example, while we found about a quarter of organizations mentioned bisphenols on their website - likely a result of bisphenol A (BPA) being a key target for advocacy campaigns- many fewer discussed air pollution (11%) or oxybenzone (7%) in spite of these being chemicals studied in the BCERP program. As the research progresses, sharing these results widely can broaden the public focus beyond a limited number of chemicals.

Focus group participants and interviewees repeatedly mentioned the importance of increased access to experts and easy-to adopt programs. To help translate study results, individual researchers and federally-funded research programs could provide breast cancer organizations with already structured educational programs to engage their communities and share online webinars, videos, and “expert talks.” Researchers could provide information to breast cancer organizations in formats that are readily adaptable to social media dissemination as many of these organizations connect with the public through platforms like Facebook and Twitter, and blogs, and social media dissemination is an effective strategy for sharing breast cancer and environmental risk information [37]. Information could also be provided at large meetings such as the San Antonio Breast Cancer Symposium. As smaller organizations often turn to larger organizations, such as the American Cancer Society and Komen, for their information, it would be strategic for researchers and federal agencies to target these influential organizations in their research translation activities.

To engage the public with emerging research findings, the websites of prominent, national groups, such as NCI and ACS, should be revised to help individuals, breast cancer organizations, and health professionals better understand the state of the evidence on environmental breast carcinogens and explain paradigms for identifying risk factors for complex, multifactorial and long-latency diseases. This would include integrating evidence from in vivo, in vitro*,* and human studies, recognizing the importance of windows of susceptibility, and considering pathways to disease such as endocrine disruption and interactions with genetic susceptibility. Websites can explain evidentiary standards that are increasingly accepted in environmental health, such as strength of evidence approaches, as well as recommend reducing exposures from a precautionary standpoint. The President’s Cancer Panel, Institute of Medicine (IOM), and IBCERCC have helpful language that could inform website content for these nationally-important groups. For example, with respect to several environmental chemicals, IOM’s report states.

“… it may be prudent to avoid or minimize exposure because the available evidence suggests biological plausibility for exposure to be associated with an increased risk of breast cancer, or there is suggestive evidence from epidemiology, or both [20].

The report further states that

Exposure to chemicals with estrogenic or other properties relevant to sex steroid activity, such as bisphenol A (BPA), polybrominated diphenyl ethers (PBDEs), zearalenone, and certain dioxins and dioxin-like compounds, may influence breast cancer risk, especially if those exposures occur at certain life stages or in combination with exposure to other similar chemicals, certain dietary components, or other factors [20].

Developing website content and other communications that reflect current scientific knowledge will require collaboration of researchers with communications specialists and community partners to ensure that the language is accessible and culturally appropriate. Some of the websites included in this study, such as the websites of Silent Spring Institute and Breast Cancer Prevention Partners, as well as websites outside this study such as BCERP, are already including this type of content for lay audiences and can be models for others [38–40]. Social media and in-person communications offer other channels for expanding public understanding.

In addition, representatives of breast cancer organizations, cited barriers that were not just scientific. Leaders were concerned about burdening economically-challenged members with environmental information, but they can be reassured that previous work found that diverse types of communities often appreciate information on potential environmental risks, and individuals will make policy and behavioral changes regardless of their socioeconomic status and linguistic backgrounds [41, 42]. It is thus helpful for communication about environmental factors to contain culturally-appropriate, actionable tips for individuals and communities across the social and economic spectrum and for materials to be translated into multiple languages when relevant.

Even if research translational activities are increased, our focus groups and interviews emphasized the structural barriers that challenge the ability of breast cancer organizations to focus on disease prevention. In particular, breast cancer organizations are often doing the essential work of providing support and services to diagnosed women who are underinsured or face other financial, educational, or linguistic barriers to receiving care. As such, many of these small, largely under-resourced groups were created to address problems with the U.S. health care system and have less time to dedicate to helping prevent the disease.

Further, as breast cancer organizations are often underfunded and resource-limited, many rely on corporate contributions to provide their programs, services, and events, with biotech and pharmaceutical companies in particular being significant sponsors. When undertaking our website analysis, we documented frequent sponsorship by companies such as Astrazeneca, Genentech, Pfizer, Tesaro, Celgene, Novartis, and Merck, who have financial interests in the diagnosis and treatment of breast cancer rather than in its prevention. This may cause breast cancer organizations to also preferentially focus on medical responses to breast cancer. In contrast, early breast cancer activists promoted research agendas that challenged the focus on screening and treatment of the disease and were attuned to policy issues, community concerns, and industrial health threats [22, 43]. Going forward, their perspectives could be amplified and strengthened by maintaining and increasing public funding for environmental research that empowers partnerships with grassroots organizations to ensure that breast cancer advocates and communities help set research agendas and translate results.

### Limitations and suggestions for future work

While we sought to use unbiased internet searches to identify breast cancer activist organizations, individuals familiar with the work of Silent Spring Institute may have been more willing to participate in interviews and focus groups, and our additional snowball sampling may also be more likely to identify participants who were acquainted with Silent Spring. Our sample may thus be skewed towards breast cancer activists who already care about environmental links to breast cancer. Despite this, even those who are concerned about potential environmental risks underscored the difficulties they face to incorporating these issues into their work and the majority of websites contained little information about specific environmental factors. The general challenge for breast cancer advocates to incorporate environmental chemicals into their work is therefore likely greater than that represented by our data, as we found even motivated advocates have difficulty figuring out action steps.

An additional limitation is that website analyses may not capture the breadth and broader context of an organization’s work. For example, a key mission of the West Islip Breast Cancer Coalition for Long Island is to raise awareness about the environment and breast cancer and their advocacy led to the multiyear Long Island breast cancer study mandated by congress in 1993. However, their website had few references to suspected environmental breast carcinogens. While the website analysis may thus underestimate the contributions of some organizations to addressing environmental factors, it also draws attention to the systemic absence of information about breast cancer and the environment on websites and highlights areas where information dissemination can be improved.

While we studied websites to select the most appropriate terms for our study, terms are sometimes used in a context that is unrelated to a breast cancer risk factor (e.g., exercise is frequently used in a preventive context, but is occasionally used to describe exercises after surgery).

While we found 40% of breast cancer organizations discuss the environment on their websites, some of this discussion discounts the environment as an important risk factor rather than informing exposure reduction from a precautionary standpoint. Kulkarni et al. [36] analyzed the most popular websites that discuss breast cancer risk and the environment and found that a significant proportion of the content (49%) suggested that there is no association between environmental exposures and breast cancer or emphasized that the research is inconclusive, most contained technical language that limited the readability of the websites, and few had information that was culturally tailored.

While our website analysis focused on U.S. breast cancer organizations, breast cancer incidence rates are increasing worldwide and future studies could assess whether breast cancer organizations abroad incorporate prevention around environmental factors into their work or if they are similarly focused in large part on services provision.

## Conclusion

Our research documents that breast cancer organizations in the U.S. have incorporated little information about potential chemical contributors to breast cancer on their websites, with prevention messages preferentially focusing on lifestyle-related risk factors. While attention to lifestyle risk factors, such as a lack of exercise and alcohol consumption, are important components of a prevention-based strategy, an overemphasis on lifestyle and genetic risk factors can overburden individuals with a sense of responsibility for their disease, while limiting their informed participation in decisions about environmental regulations, safety testing of chemicals, and personal choices that influence chemical exposures. Interviews and focus groups revealed that organizations would like assistance in incorporating environmental-based messages into their work. Federal agencies and scientists can help them surmount some of the knowledge and resource-related barriers to including environmental content by disseminating research in formats that are easy for time-strapped organizations to incorporate into existing programs and by including actionable exposure reduction tips that are culturally sensitive and mindful of financial barriers. Breast cancer organizations, however, would still face institutional obstacles to focusing on prevention-based messages, such as the downplaying of potential environmental risks by industry interests, as well as having to focus limited resources on ensuring that individuals diagnosed with breast cancer receive care and support. Thus, environmental research translation to public health should consider strategies beyond clear communications, including supporting stronger environmental regulations and informing prevention-based healthcare.

## Supplementary information

**Additional file 1: Table S1.** Organizations included in website analyses and their geographic scope.

## Data Availability

The datasets generated and analyzed during the current study, as well as the R code used for analysis, are available in the Github repository, https://github.com/breastcancer-info-research/data.

## Abbreviations

ACS: American Cancer Society
BCERP: Breast Cancer and the Environment Research Program
BPA: Bisphenol A
BRCA: BReast CAncer gene
CBCRP: California Breast Cancer Research Program
DDT: Dichlorodiphenyltrichloroethane
DES: Diethylstilbestrol
DOD: Department of Defense
EDC: Endocrine disrupting chemicals
EPA: Environmental Protection Agency
FDA: Food and Drug Administration
HRT: Hormone replacement therapy
IARC: International Agency for Research on Cancer
IBCERCC: Interagency Breast Cancer and Environmental Research Coordinating Committee
IOM: Institute of Medicine
MBCC: Massachusetts Breast Cancer Coalition
NCI: National Cancer Institute
NIEHS: National Institute of Environmental Health Sciences
NIH: National Institutes of Health
OSHA: Occupational Safety and Health Administration
PAHs: Polycyclic aromatic hydrocarbons (products of combustion)
PCBs: Polychlorinated biphenyls
PCP: President’s Cancer Panel
PFAS: Poly and perfluoroalkyl substances
PFCs: Perfluorochemicals or perfluorinated chemicals
PFOA: Perfluorooctanoic acid
PFOS: Perfluorooctanesulfonic acid
