# Supplementary material for: Barriers and opportunities for breast cancer organizations to focus on environmental health and disease prevention: a mixed-methods approach using website analyses, interviews, and focus groups
Source: Environ Health. 2020 Feb 10;19:15. doi: 10.1186/s12940-020-0570-7 (PMC7011560; doi:10.1186/s12940-020-0570-7)
Supplement: Supplementary file 1 — Additional file 1: Table S1. Organizations included in website analyses and their geographic scope. [file 12940_2020_570_MOESM1_ESM.docx]

**Additional file 1**

**Table S1.** Organizations included in website analyses and their geographic scope.

| **Organizations included in website analyses** | **Organization Type** |
| --- | --- |
| Adelphi New York Statewide Breast Cancer Hotline and Support Program | Local/regional |
| African American Breast Cancer Alliance | Local/regional |
| Alamo Breast Cancer Foundation | Local/regional |
| American Association of Breast Cancer Professionals | National |
| American Breast Cancer Foundation | National |
| Beyond Pink Team | Local/regional |
| Breast and Gyn Health Project | Local/regional |
| Breast Cancer Action | National |
| Breast Cancer Alliance | National |
| Breast Cancer Assistance Group of Monterey County | Local/regional |
| Breast Cancer Care & Research Fund | Local/regional |
| Breast Cancer Coalition of Rochester | Local/regional |
| Breast Cancer Collaborative of Texas | Local/regional |
| Breast Cancer Family Foundation | Local/regional |
| Breast Cancer Foundation of the Ozarks | Local/regional |
| Breast Cancer Network of WNY | Local/regional |
| Breast Cancer Options | Local/regional |
| Breast Cancer Prevention Partners | National |
| Breast Cancer Research Foundation | National |
| Breast Investigators | National |
| Breastcancer.org | National |
| Celebrating Life Foundation | Local/regional |
| Circulo de Vida | Local/regional |
| Coalition for Women's Cancers | Local/regional |
| Delaware Breast Cancer Coalition | Local/regional |
| Dr. Susan Love Research Foundation | National |
| Florida Breast Cancer Foundation | Local/regional |
| FORCE | National |
| Georgia Breast Cancer Coalition Fund | Local/regional |
| Great Neck Breast Cancer Coalition | Local/regional |
| Huntington Breast Cancer Action Coalition | Local/regional |
| Imaginis | National |
| Inflammatory Breast Cancer Foundation | National |
| Keep a Breast | National |
| Latinas Contra Cancer | National |
| Linda Creed Breast Cancer.Org | Local/regional |
| Links for Life | Local/regional |
| Living Beyond Breast Cancer | National |
| Long Beach Breast Cancer Coalition | Local/regional |
| Maine Breast Cancer Coalition | Local/regional |
| Manhasset Women's Coalition Against Breast Cancer | Local/regional |
| Massachusetts Breast Cancer Coalition | Local/regional |
| Men Against Breast Cancer | National |
| Metastatic Breast Cancer Alliance | National |
| Metastatic Breast Cancer Network | National |
| Minnesota Breast Cancer Coalition | Local/regional |
| Mothers Supporting Daughters with Breast Cancer | National |
| My Breast Cancer Support | Local/regional |
| National Breast Cancer Coalition | National |
| National Breast Cancer Foundation | National |
| New Hampshire Breast Cancer Coalition | Local/regional |
| Northern Ohio Breast Cancer Coalition | Local/regional |
| PA Breast Cancer Coalition | Local/regional |
| Pink Pursuit | Local/regional |
| SHARE for Women Facing Breast or Ovarian Cancer | National |
| SHARSHERET | National |
| Silent Spring | National |
| Sisters Network | National |
| Sisters Network, Chicago Chapter | Local/regional |
| Sisters Network, Dallas | Local/regional |
| Sisters Surviving Foundation | National |
| South Jersey Breast Cancer Coalition | Local/regional |
| St. Louis Breast Cancer Coalition | Local/regional |
| Susan G. Komen | National |
| Susan G. Komen, Central Virginia | Local/regional |
| Susan G. Komen, Houston | Local/regional |
| Tennessee Breast Cancer Coalition | Local/regional |
| The New York State Breast Cancer Support and Education Network | Local/regional |
| The Pink Fund | National |
| Tigerlily Foundation | National |
| To Life | National |
| Triple Negative Breast Cancer Foundation | National |
| Triple Step for the Cure | National |
| Vera Bradley Foundation for Breast Cancer | National |
| Virginia Breast Cancer Foundation | Local/regional |
| West Islip Breast Cancer Coalition for Long Island, Inc | Local/regional |
| Wisconsin Breast Cancer Coalition | Local/regional |
| Women's Cancer Resource Center | Local/regional |
| Women of Color, Breast Cancer Survivors' Support Project | Local/regional |
| Young Survival Coalition | National |
| Zero Breast Cancer | National |
